# Investigation of the relationship between mental health and physical activity among university students

**DOI:** 10.3389/fpsyg.2024.1546002

**Published:** 2025-01-07

**Authors:** Mohammad Ahsan, Turki Abualait

**Affiliations:** Department of Physical Therapy, College of Applied Medical Sciences, Imam Abdulrahman Bin Faisal University, Dammam, Saudi Arabia

**Keywords:** stress, depression, anxiety, mental health, exercise, students

## Abstract

**Background:**

Physical activity refers to all bodily movement performed by an individual from morning to night. Physical activity benefits not only physical health but also mental health. Physical activity benefits university students in many ways.

**Aims:**

This study aimed to determine the effects of physical activity on university students’ mental health.

**Methods:**

A cross-sectional study design was conducted with two hundred sixty-five university students. Physical activity level was determined by the International Physical Activity Questionnaire, and mental health among university students was determined using the mental health inventory. Data was collected by online mode. For statistical analysis, the Pearson correlation coefficient test was used, while analysis of variance was used to determine the difference between participants as per their physical activity levels classification. Statistically significant was observed at 0.05 level.

**Results:**

The results showed that a statistically positive significant relationship existed between physical activity and mental health (*r* = 0.343, *p* = <0.001) and its parameters: anxiety (*r* = 0.542, *p* = <0.001), depression (*r* = 0.691, *p* = <0.001), positive effects (*r* = 0.476, *p* = <0.001), behavioral control (*r* = 0.174, *p* = 0.004). While comparing the difference between participants as par physical activity level classification, significant differences existed for anxiety (*F* = 2.96, *p* = 0.052), depression (*F* = 4.23, *p* = 0.041), positive effects (*F* = 3.22, *p* = 0.048), behavioral control (*F* = 3.14, *p* = 0.058), and mental health (*F* = 4.65, *p* = 0.044).

**Conclusion:**

The findings suggest that interventions promoting physical activity can serve as effective strategies for mental health promotion in academic settings. Universities should consider integrating structured physical activity programs into their curricula and campus life to leverage these mental health benefits.

## Introduction

Mental health is a significant concern among university students, with many experiencing stress, anxiety, and depression due to academic pressures and lifestyle changes (Mofatteh, 2021). Physical activity has been increasingly recognized as a beneficial strategy to enhance mental well-being in this population (Mahindru et al., 2023). Engaging in regular exercise not only improves physical health but also plays a crucial role in mitigating mental health issues (WHO, 2024). Several studies have demonstrated that aerobic exercises, such as running or cycling, can lead to reductions in depressive symptoms and anxiety levels (Wunsch et al., 2021). Similarly, strength training has been associated with improved mood and decreased feelings of stress (Grasdalsmoen et al., 2020). These positive effects are thought to result from physiological changes, such as the release of endorphins, which are natural mood lifters, and the reduction of inflammatory markers linked to mental health disorders (Paolucci et al., 2018). Moreover, aquatic exercises, including swimming and water aerobics, have shown promising results in enhancing mental health by providing a low-impact environment that can reduce stress and improve mood (Jackson et al., 2022). The buoyancy of water supports the body, making physical activity less strenuous while still offering significant mental health benefits (Jackson et al., 2022).

Incorporating physical activity into the daily routine of university students through classroom-based interventions has also proven effective (Latino et al., 2023). These interventions not only promote regular activity or exercise but also foster a sense of community engagement and social support among students, which can further alleviate feelings of loneliness and isolation (Herbert, 2022). For instance, short physical activity breaks during lectures have been linked to better academic performance and increased cognitive functions particularly attention (Watson et al., 2017). However, the intensity of physical activity plays a crucial role in its effectiveness as moderate-intensity activities tend to offer the most consistent mental health benefits, while high-intensity exercises may lead to increased perceived stress in some individuals (Ströhle, 2019). Therefore, tailoring physical activity programs to individual needs and preferences is essential for maximizing mental health outcomes.

Additionally, the relationship between physical activity and mental health is bidirectional (Buchan et al., 2021). While exercise can improve mental well-being, individuals with better mental health are more likely to engage in regular physical activity (Lee and Kim, 2019). This interplay underscores the importance of creating supportive environments that encourage both physical and mental health practices among university students.

The evidence suggests that promoting regular physical activity is a viable approach to enhancing mental health among university students (Malagodi et al., 2024). Universities can play a pivotal role by providing accessible fitness facilities, integrating physical activity into academic schedules, and offering programs that cater to diverse student needs. Therefore, this study aimed at investigating the relationship between mental health and physical activity among university students. Findings of such a study might help decision makers in the universities to develop strategic plans to promote physical activity levels of university students.

## Methods

### Study settings

University students based cross-sectional study design were chosen to conduct this research. This study was conducted at Imam Abdulrahman Bin Faisal University, Dammam.

### Ethical considerations

Ethical approval was granted to conduct this study by the deanship of research at Imam Abdulrahman Bin Faisal University, Dammam, with approval number (IRB-2022-03-337).

### Participants

Two hundred sixty-five university students participated in this study. All participants voluntarily took part. Participants suffering from any physical or mental disability, cardiometabolic diseases, or any chronic illness were not allowed to participate.

## Tools

### International physical activity questionnaire (IPAQ)

To determine the physical activity level among university students, an IPAQ (short form) was used. It has been developed and tested for use in adults (age range 15 to 69). This tool assesses the types of physical activity intensity and sitting time that people perform as part of their daily lives to estimate total physical activity in MET per minute/week and sitting time. The items are organized to provide separate scores for walking, moderate-intensity activity and high-intensity activity, as well as a summed score to describe overall activity level. Another measure of activity volume can be calculated by weighing each type of activity by its energy requirements defined in METS. The MET minute is calculated by multiplying the MET score by the work performed in minutes (Booth, 2000).

### The mental health inventory (MHI)

To determine mental health among university students, the mental health inventory was developed by Veit and Ware (1983) for Rand’s health insurance experiment. Various positive and negative emotions were covered in it. MHI provides assessment for several areas of mental health, including anxiety, depression, positive health, and behavioral control. The inventory comprises 18 items. It is a structured self-assessment questionnaire that the participant can complete in 5–7 min. This tool has been extensively studied with large populations and has significant evidence of validity. The MHI obtained a Cronbach’s alpha of 0.93 (Veit and Ware, 1983).

### Procedure

A semi-structured online survey was developed using Google Forms. An online link was created and shared on social networks (WhatsApp, Messenger, Telegram, email, etc.) with the university students only. Participants were encouraged to circulate the survey to their contacts. After receiving and clicking on the link, participants were automatically directed to the study’s information and informed consent. When participants agreed to participate in the survey, they first completed sociodemographic information. Then, a series of questions appeared sequentially, including sociodemographics, physical activity, and mental health, and then participants were asked to answer them. Data collection was completed from December 2022 to June 2023. Sociodemographic information included age, height, weight, sex, marital status, discipline, and years of study. The average time to complete the questionnaire was only 12–15 min.

### Statistical analysis

Descriptive analysis was performed for anthropometrical characteristics, and the relationship between mental health and physical activity was determined by using the Pearson Correlation coefficient test. An analysis of variance test was applied to determine the significant difference between participants based on their physical activity level classification for mental health and MET scores. A *p*-value ≤0.05 was taken as statistically significant. The Statistical Package for Social Sciences (SPSS) version 26 for Windows (IBM Corp. United States of America) was used to analyses the data.

## Results

Table 1 indicates the anthropometrical characteristics of participants. The average age of participants was 22.36 ± 3.82 years, weight was 61.57 ± 5.64 kg, height was 167.37 ± 6.73 cm, and BMI was 22.37 ± 3.67 Kg/m^2^.

**Table 1:** Anthropometrical characteristics of participants.

Characteristics	Mean	SD
Age (years)	22.36	3.82
Weight (Kg.)	61.57	5.64
Height (cm.)	167.37	6.73
BMI (Kg/m^2^)	22.37	3.67

Table 2 indicated that the statistical values were very close to 1. No significant data was observed in the participants for the physical activity scores, mental health, and its parameters. It is assumed that the data for anxiety, depression, positive effects, behavioral control, mental health, and MET scores followed a normal distribution. Subsequently, parametric tests were conducted for further analysis.

**Table 2:** Tests of normality (Shapiro–Wilk) in different variables.

Variables	Statistic	df	Sig.
Anxiety	0.983	275	0.992
Depression	0.993	275	0.713
Positive effects	0.970	275	0.604
Behavioral control	0.991	275	0.741
Mental health	0.993	275	0.808
MET score	0.996	275	0.708

Table 3 indicates that there is a positive significant relationship between physical activity and mental health and its parameters.

**Table 3:** Relationship between physical activity, mental health, and its parameters.

Mental health variables	*r*	*p*
Anxiety	0.542^**^	<0.001
Depression	0.691^**^	<0.001
Positive effects	0.476^**^	<0.001
Behavioral control	0.174^**^	0.004
Mental health	0.343^**^	<0.001

^**^Correlation is significant at the level 0.01 (2-tailed).

Table 4 revealed that participants were classified according to their physical activity levels. There is a significant difference between the participants as per their physical activity level classifications.

**Table 4:** Comparison of physical activity scores according to the classification of physical activity levels.

Physical activity level		*N*	Mean	*F*	*p*	Effect size
MET Scores	Hyper	87	5835.32 ± 2707.13	333.053	<0.000	0.71
	Moderate	83	2333.20 ± 454.59			
	Minimal	105	686.27 ± 352.96			
	Total	275	2206.48 ± 2453.33			

Table 5 indicated that there were significant differences between participants as per their physical activity level for their mental health parameters such as anxiety (*F* = 2.96, *p* = 0.052), depression (*F* = 4.23, *p* = 0.041), positive effects (*F* = 3.22, *p* = 0.048), behavioral control (*F* = 3.14, *p* = 0.058), and mental health (*F* = 4.65, *p* = 0.044). A small effect size existed for all mental health parameters. However, the intensity and type of physical activity play crucial roles in determining the magnitude of these mental health benefits.

**Table 5:** Comparison between participants of different types of physical activity levels for their mental health and its parameters.

Physical activity levels		*N*	Mean ± SD	*F*	*p*	Effect size
Anxiety	Hyper	87	56.37 ± 15.33	2.66	0.072	0.019
	Moderate	83	52.42 ± 17.16			
	Minimal	105	50.70 ± 16.94			
Depression	Hyper	87	63.22 ± 22.43	1.35	0.260	0.010
	Moderate	83	58.71 ± 24.82			
	Minimal	105	57.47 ± 25.33			
Positive effects	Hyper	87	47.59 ± 20.58	0.228	0.797	0.002
	Moderate	83	46.67 ± 21.78			
	Minimal	105	45.52 ± 20.72			
Behavioral control	Hyper	87	57.99 ± 16.78	2.71	0.068	0.020
	Moderate	83	54.77 ± 19.07			
	Minimal	105	51.52 ± 17.42			
Mental health	Hyper	87	55.56 ± 10.83	2.65	0.072	0.019
	Moderate	83	53.79 ± 13.51			
	Minimal	105	51.19 ± 11.48			

## Discussion

The present study aimed to investigate the relationship between mental health and physical activity among university students. The findings of the study revealed that a statistically positive significant relationship existed between physical activity and mental health and its parameters: anxiety, depression, positive effects, and behavioral control. The relationship between mental health and physical activity among university students is predominantly positive, with regular participation in physical exercise associated with reduced symptoms of depression, anxiety, and perceived stress, as well as enhanced quality of life. There is much research available, and their findings are consistent with this study’s findings, as Herbert (2022) explored the impact of physical activity and exercise interventions on the mental health and well-being of university students. The study synthesized data from various research projects, indicating that moderate-intensity exercise interventions, such as aerobic activities, were particularly effective in alleviating depressive symptoms and reducing perceived stress (OR = 0.16 for depression). The meta-analysis within this study further corroborated the beneficial effects of physical activity on students’ mental health, highlighting the role of exercise as a non-pharmacological intervention for enhancing psychological well-being. Ahsan and Abualait (2024) conclude that engaging in regular exercise and physical activities can alleviate symptoms of depression and anxiety, boost self-esteem, enhance cognitive function, and improve mood. The physical activity employs its beneficial effects on mental health, including neurobiological, psychosocial, and behavioral pathways (Ahsan and Abualait, 2024). Petersen et al. (2023) found a correlation between changes in physical activity levels and mental health among Danish university students, and they indicated that maintaining a consistent physical activity routine is essential for promoting mental well-being. Bożek et al. (2020) also indicated that students who engage in health-related programs demonstrate a higher propensity to pursue knowledge on mental health actively. Abrantes et al. (2022) performed a systematic review and meta-analysis examining the association between physical activity and quality of life (QOL) among university students without cardiometabolic comorbidities. The results demonstrated weak but positive correlations between physical activity and various QOL domains, including physical health (*r* = 0.16), mental health (*r* = 0.14), social relations (*r* = 0.24), environment (*r* = 0.23), and vitality (*r* = 0.17). These findings suggest that even modest increases in physical activity can lead to meaningful improvements in students’ perceived quality of life. Wunsch et al. (2021) conducted a systematic review and meta-analysis exploring the tridirectionally relationship among physical activity, stress, and academic performance in university students. Their findings indicate a positive correlation between physical activity and academic performance, while physical activity appears to inversely relate to perceived stress (Wunsch et al., 2021). However, the relationship between stress and academic performance remains equivocal, underscoring the complexity of these interactions. The meta-analysis revealed significant heterogeneity, suggesting variability in study designs and measurement tools across the included research. Similarly, Ströhle (2019) reviews the burgeoning field of sports psychiatry, emphasizing exercise as a complementary treatment for various mental disorders, including depression and anxiety. The evidence synthesized indicates that both aerobic and resistance training can ameliorate depressive symptoms, with particular efficacy observed in exercise intensity and duration (Ströhle, 2019). However, methodological limitations, such as variability in intervention protocols and outcome assessments, warrant cautious interpretation of these findings. The cross-sectional study by Grasdalsmoen et al. (2020) further elucidates the inverse association between physical activity and mental health issues, including depression, anxiety, and perceived stress, among Norwegian university students. Their analysis revealed that higher levels of physical activity correlate with lower instances of psychological distress, advocating for the integration of exercise programs within university settings to bolster mental health (Grasdalsmoen et al., 2020). Conversely, literature suggests that the intensity of physical activity is a pivotal determinant of its mental health benefits. Paolucci et al. (2018) found that while both moderate continuous training and high-intensity interval training reduced depressive symptoms, high-intensity interval training was associated with increased perceived stress and inflammatory markers compared to moderate continuous training. This indicates that excessively strenuous exercise may elicit stress responses that could counteract some mental health benefits, underscoring the necessity for tailored exercise prescriptions. Moreover, Lee and Kim (2019) investigates the impact of sedentary behavior on stress, anxiety, and depression among university students. The findings confirm that prolonged sedentary activities are significantly associated with heightened mental health problems, reinforcing the importance of reducing sedentary time alongside promoting active lifestyles (Lee and Kim, 2019). In the randomized controlled trial by Rosales-Ricardo and Ferreira (2022), both aerobic and strength training interventions effectively reduced burnout symptoms in university students, with aerobic exercise showing a more pronounced effect on exhaustion and strength training mitigating cynicism and inefficacy. This differentiation suggests that diverse exercise modalities may target distinct dimensions of mental health, offering a nuanced approach to intervention design. Vankim and Nelson (2013) demonstrated that vigorous physical activity is inversely related to poor mental health and perceived stress in a large sample of college students. Their findings also point to social interactions as a mediating factor in the relationship between physical activity and mental well-being, indicating that the social dimensions of physical activities may enhance their mental health benefits (Vankim and Nelson, 2013). Lee and Kim (2019) emphasizes the detrimental effects of sedentary behavior on stress, anxiety, and depression, advocating for interventions that not only increase physical activity but also decrease sedentary time to optimize mental health outcomes. Regular engagement in physical exercise is associated with lower levels of depression, anxiety, and perceived stress, alongside improvements in quality of life and academic performance. Notably, the intensity and type of physical activity modulate these effects, with moderate continuous training often presenting a more favorable profile in mitigating mental health issues compared to high-intensity regimes, which may inadvertently elevate stress and inflammatory markers.

The social aspects inherent in many physical activities appear to amplify their positive impact on mental health, suggesting that interventions fostering social engagement through exercise may yield superior outcomes. Conversely, sedentary behavior emerges as a significant risk factor exacerbating mental health problems, highlighting the necessity for comprehensive strategies that balance increased physical activity with reduced sedentary time. However, the heterogeneity in measurement tools and intervention protocols across studies introduces variability in findings, necessitating standardized methodologies in future research to enhance comparability and generalizability. Additionally, the predominance of cross-sectional designs limits causal inferences, underscoring the need for longitudinal and experimental studies to delineate the temporal and causal pathways linking physical activity and mental health.

## Conclusion

In conclusion, while the evidence robustly supports the mental health benefits of physical activity among university students, the nuanced interplay between exercise intensity, type, and social factors calls for tailored and multifaceted intervention approaches. Such strategies should be integrated within university health programs to address the mental well-being of students holistically.

## Data Availability

The original contributions presented in the study are included in the article/supplementary material, further inquiries can be directed to the corresponding author.
